# Acupuncture as an Adjunct to Physiotherapy in Post-Stroke Spastic Hand: Comparative Case Series Analysis

**DOI:** 10.7759/cureus.105930

**Published:** 2026-03-26

**Authors:** Stephen C Fernand, Thomas Friedemann, Shikay Loong, Haihan Zhang, Sven Schröder, Weidong Pan

**Affiliations:** 1 Department of Traditional Chinese Medicine, Hanse Merkur Center of Traditional Chinese Medicine, University Medical Center Hamburg-Eppendorf, Hamburg-Eppendorf, DEU; 2 Department of Traditional Chinese Medicine, Shuguang Hospital Affiliated to Shanghai University of Traditional Chinese Medicine, Shanghai, CHN; 3 Department of Neurology Traditional Chinese Medicine, Anhui Branch, Shuguang Hospital Affiliated to Shanghai University of Traditional Chinese Medicine, Shanghai, CHN; 4 Department of Neurology, Shanghai University of Traditional Chinese Medicine, Shanghai, CHN; 5 Department of Encephalopathy, Shanghai Pudong New Area Hospital, Affiliated to Shanghai University of Traditional Chinese Medicine, Shanghai, CHN; 6 Department of Neurology, Shuguang Hospital Affiliated to Shanghai University of Traditional Chinese Medicine, Shanghai, CHN; 7 Department of Neurology Traditional Chinese Medicine, Shugang Hospital, Shanghai, CHN

**Keywords:** acupuncture, cerebral infarction, motor function improvement, rehabilitation, spasticity, stretching, stroke, upper-limb motor recovery

## Abstract

Background: Post-stroke hand spasticity is a major contributor to persistent upper-limb disability and may limit the effectiveness of conventional physiotherapy. Acupuncture has been proposed as a neuromodulatory adjunct to enhance motor recovery and pain control; however, evidence from routine clinical practice remains limited. This retrospective case series investigated whether adding acupuncture to physiotherapy-based stretching results in superior functional outcomes compared with physiotherapy alone in patients with post-stroke spastic hand syndrome.

Methods: Clinical records of 13 adult stroke patients treated between 2022 and 2023 were retrospectively analyzed. Six patients received combined acupuncture and physiotherapy stretching (Group A), while seven received physiotherapy stretching alone (Group B). Acupuncture was administered twice weekly for eight weeks at Shixuan (EX-UE11), SI3, and SI4, followed immediately by standardized passive hand stretching. Outcomes were assessed using the Fugl-Meyer Assessment for the Upper Extremity (FMA-UE), including total score and subdomains (motor function, joint pain, passive joint motion, and sensation). Longitudinal changes were analyzed using linear mixed-effects models, with within- and between-group comparisons of change scores.

Results: From baseline to week eight, the intervention group demonstrated a significantly greater improvement in the FMA-UE motor function subscale compared with the control group (14.3 ± 4.8 vs. 7.6 ± 6.3; *p* < 0.05). Significant between-group differences were also observed for the hand subscale (2.7 ± 1.0 vs. 0.9 ± 1.2; *p* < 0.05) and the joint pain subscale (5.0 ± 4.1 vs. 0.1 ± 5.3; *p* < 0.05). The total FMA-UE score increased significantly more in the intervention group than in the control group over the eight-week intervention period (25.5 ± 12.3 vs. 14.3 ± 8.0; *p* < 0.05). No statistically significant (ns) between-group differences were identified for the upper extremity, wrist, coordination/speed, passive joint motion, or sensation subscales (*p* > 0.05). Within-group analyses revealed significant improvements from baseline to week eight in the intervention group for motor function, hand, joint pain, and total FMA-UE (all *p* < 0.05), whereas changes observed in the control group were smaller and less consistent across subscales.

Conclusion: In this retrospective clinical analysis, acupuncture administered prior to physiotherapy stretching was associated with greater improvements in overall upper-limb motor recovery and joint pain reduction compared with physiotherapy alone in patients with post-stroke hand spasticity. These findings suggest that acupuncture may enhance conventional rehabilitation by reducing pain and facilitating motor relearning. Given the small sample size and non-randomized design, results should be interpreted cautiously. Prospective, adequately powered controlled trials are warranted to confirm efficacy and define optimal treatment protocols.

## Introduction

Stroke is the third leading cause of death worldwide, responsible for 7.3 million deaths and 160.5 million disability-adjusted life years (DALYs) in 2021 (GBD 2021 Stroke Risk Factor Collaborators). Ischemic stroke accounts for 65.3% of cases, while intracerebral and subarachnoid hemorrhages represent 28.8% and 5.8%, respectively [1]. Post-stroke motor impairments, especially upper-limb dysfunction, significantly reduce independence and quality of life, with common issues including hemiparesis, spasticity, and impaired coordination [2,3].

Spasticity, a major complication, stems from disrupted motor pathways and leads to increased muscle tone, exaggerated reflexes, and involuntary contractions. If left untreated, it may cause joint contractures and musculoskeletal deformities [4]. Despite intensive rehabilitation, 30-40% of stroke survivors continue to experience motor deficits [5,6]. The effectiveness of conventional physiotherapy is limited by factors such as spasticity, chronic pain, and variable patient responses.

Oral medications like baclofen and tizanidine frequently result in sedation and cognitive impairment, posing challenges for long-term use, particularly in elderly patients. Botulinum toxin offers temporary relief but requires repeated injections and may cause muscle weakness or localized pain. Neuromodulation and surgical interventions (e.g., deep brain stimulation, selective dorsal rhizotomy) show promise but are costly, invasive, and not suitable for all patients. Furthermore, rehabilitation requires long-term commitment and access to specialized care, which may not be feasible for everyone. Given that current treatments primarily manage symptoms rather than offer a cure, adjunctive therapies are increasingly being investigated. Emerging advances in regenerative medicine, personalized neuromodulation, and robotic-assisted rehabilitation may help address these gaps in the future [7]. Accordingly, a key objective of modern stroke rehabilitation strategies is to induce neural plasticity to facilitate and optimize neuromodulation.

Evidence suggests that neural plasticity reduces spasticity, supports muscle relaxation, and complements conventional rehabilitation [8]. Specifically, the Shixuan points (EX-UE 11), located at the tips of the 10 fingers, were selected for their traditional use in acute neurological presentations, particularly post-stroke motor dysfunction. This choice is consistent with contemporary clinical evidence that acupuncture can alleviate post-stroke spasticity [9]. Acupuncture, a central pillar of Traditional Chinese Medicine (TCM), has shown potential in stroke rehabilitation by enhancing neuroplasticity, promoting neurotransmitter release, and improving motor function [10]. Unlike standard acupuncture points, Shixuan points function as highly reactive neurovascular sites at the fingertips, where dense sensory innervation enables particularly strong afferent stimulation and downstream neuromodulatory effects [11]. Due to their placement at highly innervated fingertips, these points are particularly suitable for addressing fine motor impairments and upper-limb spasticity [12].

## Materials and methods

This study focused specifically on individuals presenting with post-stroke upper-limb spasticity, particularly cases characterized by spastic hand syndrome. The clinical profile of these patients typically included muscle stiffness, reduced voluntary control, and involuntary contractions, significantly impairing hand function [12].

Participants comprised adult patients diagnosed with spastic hand syndrome or spastic hemiparesis primarily affecting the hand following an ischemic or hemorrhagic stroke. Diagnoses were confirmed using computed tomography (CT) and/or magnetic resonance imaging (MRI) to identify cerebral infarction or hemorrhagic lesions. 

Inclusion criteria

Patients documented in medical records between 2022 and 2023 were included. Eligible participants were those presenting with spastic hand syndrome or spastic hemiparesis with predominantly spastic hand involvement, confirmed by computed tomography (CT) and/or magnetic resonance imaging (MRI), resulting from either ischemic or hemorrhagic lesions. Patients must have been undergoing routine physiotherapy for spastic hand syndrome, with some also receiving acupuncture in conjunction with physiotherapy as part of their treatment. A minimum of 30 days must have elapsed since the onset of stroke. The study included patients aged between 18 and 80 years, for whom FMA-UE scores were documented.

Exclusion criteria

Patients with spasticity caused by factors other than stroke, including conditions such as multiple sclerosis or trauma to the brain or spinal cord, were excluded. Patients with severely unstable clinical disorders were also excluded.

Intervention

**Figure 1:**
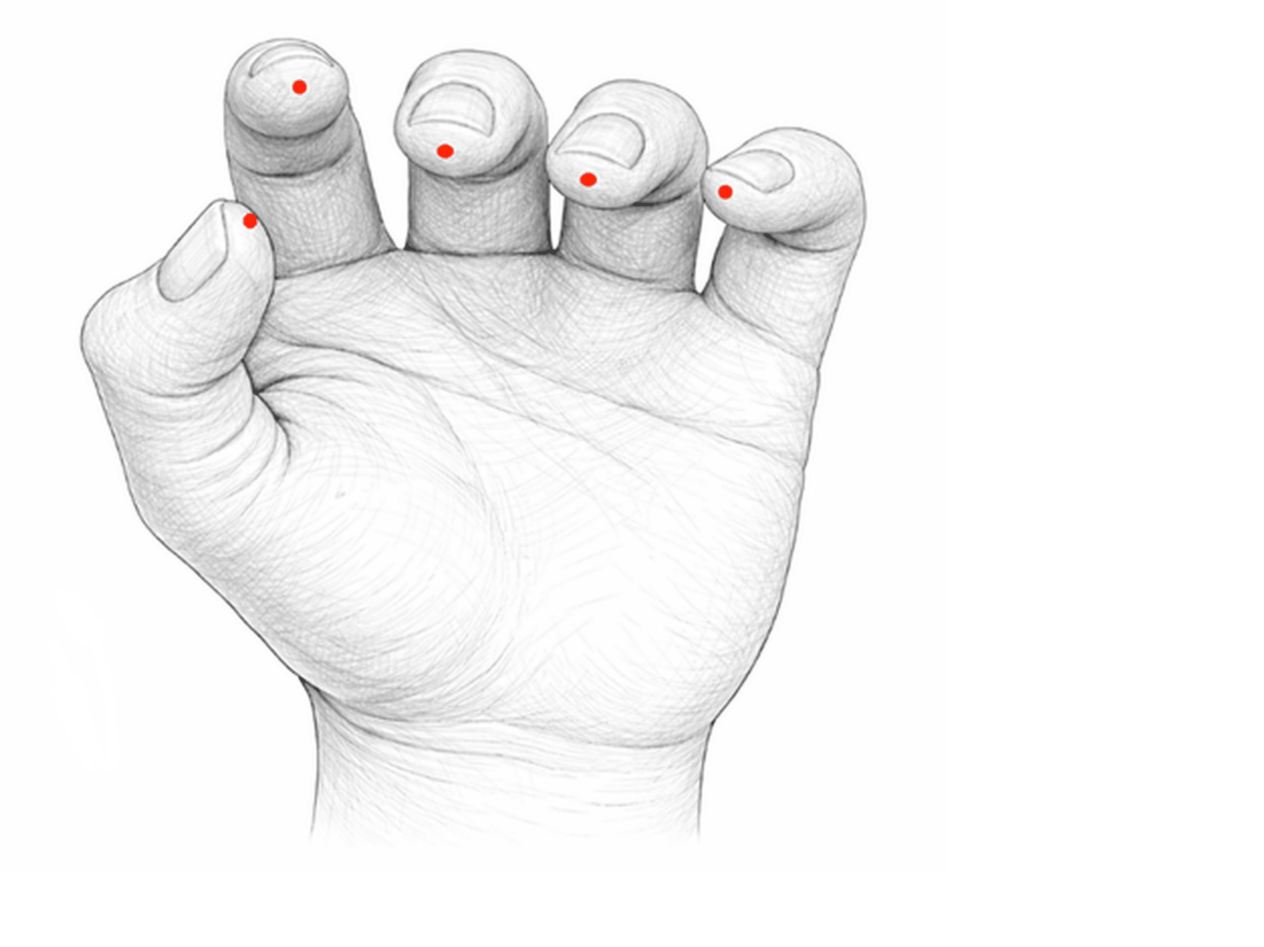
Provides a visual representation of the 5 Shixuan points (EX-UE11), illustrating their location at the fingertips for precise identification and application Original, author-designed schematic illustration of the five Shixuan points (EX-UE11) located at the palmar distal fingertips. The figure was independently conceptualized and created by the author and is intended to facilitate clear visualization of point localization for accurate identification and clinical application. This illustration is neither a reproduction nor an adaptation of official or standardized reference images.

In this retrospective, practice-based study, all consecutive patients attending their routine appointments were recorded and classified ex post into two groups: Group A received acupuncture and physiotherapy with passive stretching, and Group B received physiotherapy with passive stretching only. In addition to passive stretching exercises guided by a physiotherapist, patients in Group A received manual acupuncture treatment twice a week (≤16 sessions) for eight weeks. The treatments were performed by licensed physiotherapists with certified acupuncture training. The acupuncture modality consisted of manual needle stimulation without electrical stimulation. Sterile, single-use stainless steel needles (approximately 0.25 mm × 25-40 mm) were inserted at predefined bilateral points SI3 (Houxi), SI4 (Wangu), and Shixuan (EX-UE11). Needles were inserted to a clinically appropriate depth, according to anatomical location, and manually stimulated to elicit deqi sensation, where applicable. Needles were retained for the duration of the treatment session. Figure 1 presents 5 Shixuan points. 

Stretching was performed by a licensed physiotherapist experienced in neurological rehabilitation, following the standard institutional stretching procedures used in the rehabilitation setting. Each session lasted approximately 25 minutes and was conducted twice weekly over an eight-week period. The stretching technique involved slow, controlled movements toward an individually tolerated end range (approximately 5-10 seconds), followed by static hold positions of 30-60 seconds, with three repetitions per movement direction and rest intervals of 30-60 seconds between repetitions. Stretch intensity was maintained within a mild to moderate range, avoiding rapid movements to minimize stretch reflex activation or clonus, and proximal stabilization of the limb was ensured throughout the procedure.

To ensure consistency of the intervention, all stretching sessions were delivered by the same licensed physiotherapist. Treatment adherence was verified retrospectively through routine clinical documentation recording the number and duration of completed sessions.

In Group A, passive hand stretching was performed immediately after acupuncture during the same treatment visit, whereas Group B received passive stretching alone. Both groups attended two treatment sessions per week over a total duration of eight weeks. Because this study represents a retrospective analysis of routine clinical practice, group allocation was non-randomized and based on the treatment received. During the study period, patients continued their usual medical management and standard outpatient care as prescribed by their treating physicians; however, no additional structured upper-limb rehabilitation programs beyond the documented physiotherapy sessions were introduced.

The legal basis for conducting this retrospective analysis is provided by the Health Data Protection Act of North Rhine-Westphalia (Gesundheitsdatenschutzgesetz NRW, GDSG NRW). According to §6(2) GDSG NRW, the use of patient data for scientific research is permissible without explicit patient consent if the data are accessed by the practitioner as part of their professional duties in patient care, the research purpose cannot reasonably be achieved otherwise, and there is an overriding public interest. Furthermore, the data must be anonymized as soon as the research objective allows.

Data analysis

The data were collected in accordance with the inclusion/exclusion criteria from the physiotherapy clinic charts during 2022-2023. A total of 13 patients were identified during this period, and their outcomes were extracted and analyzed anonymously.

Measurements

The Fugl-Meyer Assessment (FMA) is a standardized and widely used instrument for assessing sensorimotor impairment following a stroke [13]. It comprises separate subscales for the upper extremity (FMA-UE) and the lower extremity (FMA-LE), with standardized administration and scoring guidelines publicly available from the University of Gothenburg, Sweden, and free to use in clinical or research settings for non-commercial purposes [14]. In the present study, upper-extremity and hand function were evaluated using the 66-point Upper Extremity subscale (FMA-UE) as the primary outcome measure to assess the effect of acupuncture as an adjunct to physiotherapy in individuals with post-stroke spasticity. The FMA-UE is a well-validated and widely recognized measure in stroke rehabilitation research, designed to quantify motor recovery of the paretic upper limb in patients with hemiparesis [15].

The upper extremity component comprises 66 items for motor function (4 subscores: Upper extremity (36 items), Hand (10 items), Wrist (14 items), Coordination/Speed (6 items), 6 items for sensation, 12 items for passive joint motion, and 12 items for joint pain, each rated on a three-point ordinal scale: 0 = inability to perform the movement, 1 = partial performance, and 2 = complete execution. The cumulative score ranges from 0 to 132 points, with higher scores signifying greater motor proficiency in the affected limb. Assessments were conducted before and after the intervention, enabling the objective quantification of functional changes over time. The use of this standardised outcome measure allowed for systematic evaluation of motor recovery and facilitated robust comparisons between the intervention and control groups. Thus, the FMA-UE provided a reliable and comprehensive framework for assessing the effectiveness of the combined therapeutic approach in enhancing upper-limb function following stroke.

Primary and secondary efficacy measures

The primary efficacy measure of the study was the improvement in FMA-UE total score. Secondary efficacy measures included the FMA-UE subcomponents (joint pain, passive joint motion, sensation, and motor function).

Statistical analysis

Statistical analyses were performed using jamovi (version 2.6, The jamovi project). Continuous variables are presented as mean ± standard deviation (SD) when normally distributed and as median with interquartile range (IQR) when the assumption of normality is not met.

A total of 13 participants were included in the analysis, with six patients in the acupuncture plus physiotherapy Group A and seven patients in the physiotherapy-only Group B.

The median value for the outcome variable was 45.5 in the acupuncture plus physiotherapy group and 60.0 in the physiotherapy-only group. This indicates that the central tendency of the measured variable was higher in the physiotherapy-only group compared with the combined treatment group.

The interquartile range (IQR), which reflects the dispersion of the middle 50% of the data, was 56.8 in the acupuncture plus physiotherapy group and 29.5 in the physiotherapy-only group. The larger IQR observed in the combined treatment group suggests greater variability among participants, whereas the physiotherapy-only group showed a more homogeneous distribution of values. 

Normality was assessed using the Shapiro-Wilk test. For normally distributed data, within-group comparisons (pre- vs. post-intervention) were conducted using paired t-tests, and between-group differences were analyzed using independent-samples t-tests. For non-normally distributed data, the Wilcoxon signed-rank test was used for within-group comparisons and the Mann-Whitney U test for between-group comparisons. Longitudinal changes in the FMA-UE total score (primary outcome) were additionally analyzed using linear mixed-effects models (LMMs). Time was treated as a continuous variable (0, 4, and 8 weeks). Fixed effects included time, group, and the group × time interaction. A random intercept was specified for each participant to account for within-subject correlation. Models were estimated to use restricted maximum likelihood (REML). A p-value < 0.05 was considered statistically significant. The FMA-UE total score was defined as the prespecified primary outcome. Additional analyses of secondary outcomes were considered exploratory and were interpreted cautiously.

Group distribution

**Figure 2:**
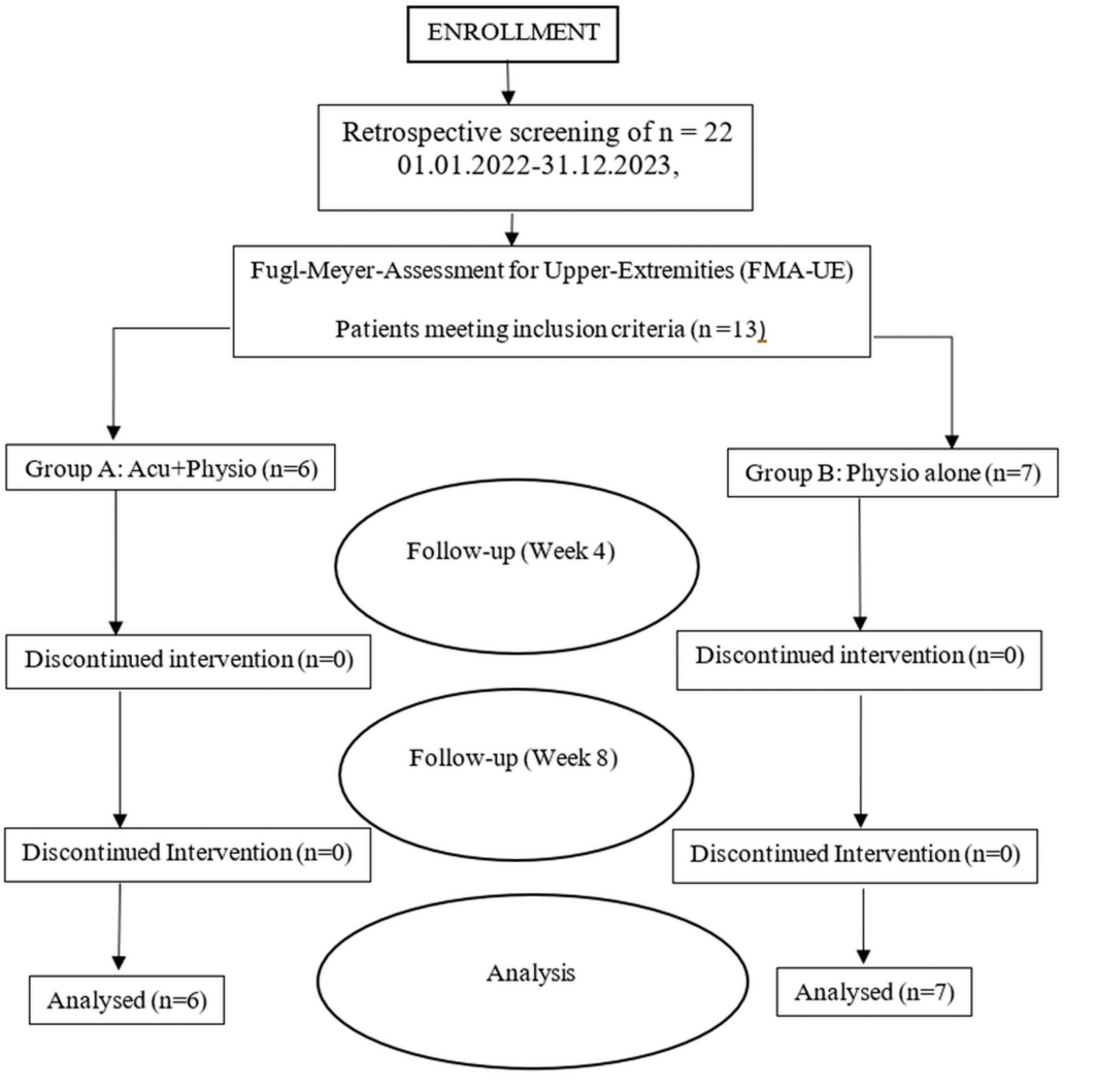
Flow diagram of participant enrollment and outcome assessments in a non-randomized study of acupuncture and stretching for post-stroke spastic upper limb motor dysfunction

The participants were divided into two groups. Group A comprised six patients who received acupuncture combined with stretching: two females and four males. Group B, which consisted of seven patients who underwent stretching alone, included seven participants. All participants completed the trial, resulting in 100% retention and adherence to the intervention protocol. Figure 2 shows the participant enrollment and analysis flow chart of the study.

## Results

Basic demographic data, such as age, sex distribution, and baseline motor function scores, were similar across groups, although detailed statistical comparisons were not provided. Secondary outcome analyses should be interpreted as exploratory.

Baseline characteristics of participants in the intervention group (acupuncture plus physiotherapy) and the control group (physiotherapy only). A total of 13 participants were included in the analysis, with six individuals allocated to the intervention group receiving acupuncture in addition to physiotherapy and seven individuals allocated to the control group receiving physiotherapy alone. The mean age was comparable between groups, with a mean age of 68.5 ± 7.6 years in the intervention group and 68.3 ± 7.3 years in the control group. In the intervention group, four participants were male (66.6%), and two were female (33.3%), while the control group consisted of five males (71.4%) and two females (28.6%). The mean time since stroke was 90.5 ± 139.7 months in the intervention group and 61.6 ± 25.4 months in the control group. Table 1 summarizes the baseline characteristics.

**Table 1:** Baseline characteristics of participants in the intervention group (acupuncture plus physiotherapy, Group A) and the control group (physiotherapy only, Group B). Data are presented as number (n) %, Mean ±SD (standard deviation).

Characteristics	Intervention Group A (Acu+Physio, n = 6)	Control Group B (Physio only, n = 7)
Age, years (mean ± SD)	68.5 ± 7.6 (mean ± SD)	68.3 ± 7.3(mean ± SD)
Male (n)	4 (66.6%)	5 (71.4%)
Female (n)	2 (33.3%)	2 (28.6%)
Time Since Stroke (month)	90.5 ± 139.7(mean ± SD)	61.6 ± 25.4(mean ± SD)

FMA-UE total score

Both groups showed improvement over eight weeks, with the acupuncture + physiotherapy Group A improving by 25.3 points and the physio Group B by 14.5 points. Although Group A showed consistently higher scores at each time point, the cross-sectional differences did not reach statistical significance (p > 0.05 at all-time points).

From baseline to week eight, the acupuncture + physiotherapy Group A showed a mean improvement of 25.5 ± 12.3 points (paired t-test, t=3.610, p <0.005). The physiotherapy-only Group B demonstrated a mean improvement of 14.3 ± 8.0 points (paired t-test, t=3.660, p < 0.005). The between-group difference in mean change was 9.9 points, indicating a significantly greater improvement in Group A (t = 2.349, p < 0.05). The time × group interaction was statistically significant (β = −1.38 points/week, SE = 0.65, p = 0.033), indicating a significantly steeper recovery trajectory in Group A compared with Group B. Over the eight-week treatment period, this corresponds to an estimated 11.04-point (8 × 1.38) greater improvement in the FMA-UE total score in the acupuncture + physiotherapy Group A relative to the physiotherapy-only Group B.

FMA-UE subscore

FMA-UE Motor Function Score

Cross-sectional comparisons showed no significant between-group differences at any time point (all p > 0.05).

From baseline to week eight, the acupuncture + physiotherapy Group A showed a mean improvement of 14.3 ± 4.8 points on the FMA-UE Motor Function subscore (paired t-test t=4.513, p <0.001). The physiotherapy-only Group B demonstrated a mean improvement of 7.6 ± 6.3 points (paired t-test, t = 2.576, p < 0.05). The between-group difference in mean change was 6.8 points, indicating a significantly greater improvement in Group A (t = 2.532, p < 0.05).

FMA-UE Motor Function-Upper Extremity Subscale

Both groups showed comparable improvements, with no significant between-group differences at any time point (all p > 0.05).

From baseline to week eight, the acupuncture + physiotherapy Group A showed a mean improvement of 6.5 ± 2.9 points on the FMA-UE motor function subscore (paired t-test t=3.858, p <0.005). The physiotherapy-only Group B demonstrated a mean improvement of 5.9 ± 1.9 points (paired t-test t=5.582, p < 0.001). Although the rate of improvement was numerically 0.6 points higher in the acupuncture plus physiotherapy group (A), the between-group difference in recovery slopes did not reach statistical significance (p = ns).

FMA-UE Motor Function Score-Hand Subscale

Cross-sectional comparisons showed no significant between-group differences at any time point (all p > 0.05).

From baseline to week eight, the acupuncture + physiotherapy Group A showed a mean improvement of 2.7 ± 1.0 points (paired t-test t=4.217, p <0.005). The physiotherapy-only Group B demonstrated a mean improvement of 0.9 ± 1.2 points (paired t-test t=1.731, p > 0.05). The improvement was numerically 1.6 points higher in the acupuncture plus physiotherapy Group A, and the between-group difference reached statistical significance (t =3.251, p<0.01).

FM-UE Motor Function-Wrist Subscale

Group A showed significant within-group improvement (4.0 ± 1.8 points, p <0.01), while Group B’s improvement did not reach significance (2.0 ± 2.3 points, p>0.05). The difference between-group was not statistically significant (p>0.05).

FM-UE Motor Function-Coordination/Speed Subscale

Group A showed numerical improvement (1.2 ± 1.2 points), while Group B showed a mean deterioration (-1.1 ± 3.9 points), though neither the within-group change nor the between-group difference reached statistical significance (all p>0.05).

FMA-UE Passive Joint Motion Score

Cross-sectional comparisons showed consistently higher scores in Group A (2.1-2.8 points), but these differences were not statistically significant (all p >0.05).

From baseline to week eight, the acupuncture + physiotherapy Group A showed a mean improvement of 4.7 ± 4.4 points on the FMA-UE passive joint motion subscore (paired t-test, t = 2.233, p <0.05). The physio-only Group B demonstrated a mean improvement of 4.3 ± 2.9 points (paired t-test, t=3.215, p < 0.01). The trajectories did not differ between groups (p = 0.841).

FMA-UE Joint Pain Score

Group A showed progressive improvement across all timepoints, while Group B remained relatively stable. The between-group difference in the FMA-UE joint pain subscore was 2.7 points at baseline in favor of Group A at baseline (t-test for independent samples, t=1.386, p>0.05), increased to 5.9 points at week four (t-test for independent samples t=2.971, p <0.05), and further increased to 7.6 points at week eight (t-test for independent samples, t=3.179, p <0.005), reaching statistical significance at weeks four and eight.

From baseline to week eight, the acupuncture + physiotherapy Group A showed a mean improvement of 5.0±4.1 (p<0.05) and of 0.1±5.3 for physio alone (B) (p =ns). The time × group interaction was statistically significant (β = -0.61 points/week, SE = 0.26, p < 0.05), corresponding to an estimated 4.9-point additional improvement over eight weeks compared with the physio-alone Group B.

FMA-UE Sensation Score

Group B showed numerically higher scores at all timepoints (0.8-1.9 points), though these differences were not statistically significant (all p >0.05).

From baseline to week eight, the acupuncture + physiotherapy Group A showed a mean improvement of 1.5±2.9 (p = ns) and of 2.4±2.5. There was no evidence of a differential effect between groups (t-test for independent samples, t= 0.232, p >0.05). 

**Figure 3:**
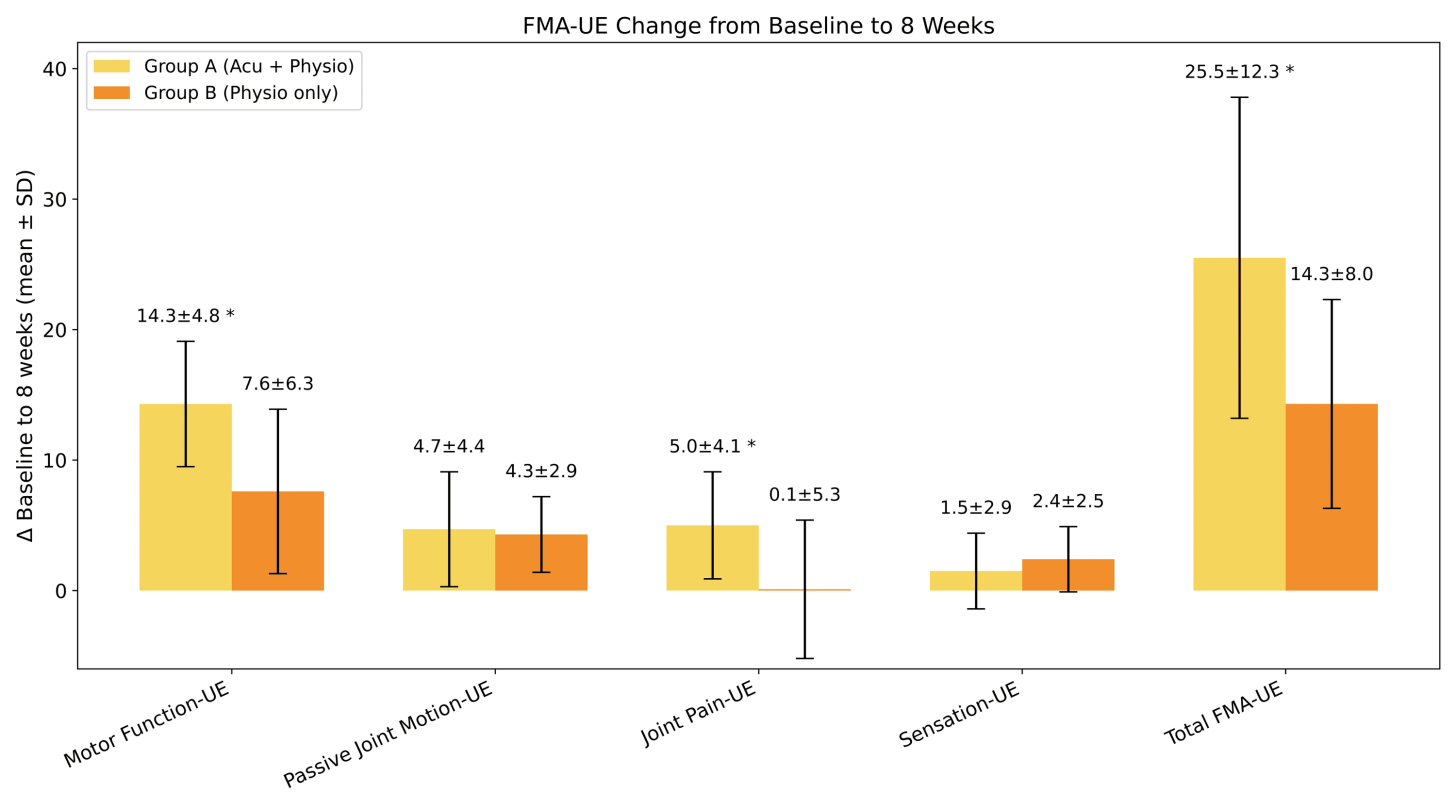
Comprehensive comparison mean improvement p-values (*= p < 0.05) FMA-UE motor-function, FMA-UE joint pain, FMA-UE passive

Table 2 and Figure 3 summarize the comparison of the FMA-UE scores.

**Table 2:** Results-scores after eight weeks: FMA-UE motor function, FMA-UE joint pain, FMA-UE passive motion, FMA-UE sensation, and FMA-UE total score. Data presented as n (number), mean ± SD (standard deviation). P-values of p<0.05 and p<0.005 are considered significant; p values of >0.05 are considered as not significant (ns). Within-group changes were analyzed using paired t-tests; between-group differences and longitudinal effects using independent t-tests; longitudinal effects of the primary outcome Total FM-UE were confirmed using linear mixed-effects models. *indicates that the Shapiro-Wilk test revealed a statistically significant deviation from normal distribution; therefore, group comparisons were performed using the Mann-Whitney U test.

FMA-UE	Group A (n=6) / B (n=7)	Baseline mean ± SD	p-value	4 weeks mean ± SD	p-value	8 weeks mean ± SD	p-value	Baseline to 8 weeks mean ± SD	Δ Baseline to 8 weeks mean ± SD	p-value
Motor Function-UE	Acu+Physio (A)	28.0±14.2	ns	30.8±14.2	ns*	42.3±17.9	ns	<0.001	14.3.±4.8	<0.05
	Physio alone (B)	31.0±10.4		29.3±12.6		38.6±15.7		<0.05	7.6±6.3	
Passive Joint Motion-UE	Acu+Physio (A)	13.5±4.9	ns	15.5±5.6	ns	18.2±5.6	ns	<0.05	4.7±4.4	ns
	Physio alone (B)	11.4±3.5		12.7±4.0		15.7±4.4		<0.05	4.3±2.9	
Joint Pain-UE	Acu+Physio (A)	11.3±5.9	ns	14.2±4.3	<0.05	16.3±6.7	<0.05	<0.05	5.0±4.1	<0.05
	Physio alone (B)	8.6±5.2		8.3±3.9		8.7±2.5		ns	0.1±5.3	
Sensation-UE	Acu+Physio (A)	6.5±4.0	ns	7.5±3.8	ns	8.0±3.3	ns*	ns	1.5±2.9	ns*
	Physio alone (B)	7.4±2.5		8.3±3.1		9.9±2.7		<0.05	2.4±2.5	
Total FMA-UE	Acu+Physio (A)	59.5±23.1	ns*	68.0±24.4	ns*	84.8±29.8	ns	<0.005	25.5±12.3	<0.05
	Physio alone (B)	58.4±17.7		58.6±21.2		72.9±20.9		<0.005	14.3±8.0	

Mean change (Δ) from baseline to eight weeks in Fugl-Meyer assessment for upper extremities (FMA-UE) subdomains for Group A (n=6) and Group B (n=7). Data are presented as mean±SD (standard deviation). Error bars represent standard deviation. Asterisks (*) indicate statistically significant between-group differences (p < 0.05). No asterisk is considered significant. Significant improvements were observed in FMA-UE Joint Pain and FMA-UE Total score for the acupuncture + physiotherapy Group A compared to the physio alone Group B between-group differences and longitudinal effects using independent t-tests; longitudinal effects of the primary outcome were confirmed using linear mixed-effects models.

## Discussion

This retrospective analysis indicates that acupuncture combined with physiotherapeutic stretching yields clinically meaningful advantages over stretching alone for post-stroke upper extremity motor function. Patients receiving the combined intervention demonstrated greater improvements in FMA-UE total and motor function scores as well as significant reductions in joint pain, whereas passive joint motion and sensory function improved similarly in both groups. These findings are consistent with previous evidence indicating that the addition of acupuncture to treatment can improve motor recovery in the upper limbs after a stroke. However, earlier meta-analyses have reported heterogeneity in study protocols and overall study quality. [16]. The marked analgesic effect in our cohort mirrors the robust pain reductions observed in the individual patient data meta-analysis by Vickers et al. [17], reinforcing the view that acupuncture offers clinically significant analgesia beyond placebo effects.

The neurophysiological mechanisms underpinning these improvements are consistent with contemporary models of post-stroke spastic paresis and upper motor neuron syndrome. Spasticity, as one component of this syndrome, arises from disrupted corticospinal inhibition, re-emergent reticulospinal dominance, and spinal hyperreflexia, all of which contribute to impaired voluntary motor control rather than solely reflecting increased muscle tone [4,18]. Experimental work on muscle tone indicates that acupuncture can modulate these pathological circuits by reducing gamma-motoneuron excitability, enhancing presynaptic inhibition of 1a afferents, and engaging descending inhibitory systems involving the periaqueductal gray and rostroventral medulla [19,20]. Such multimodal neuromodulatory effects may influence both reflex hyperexcitability and motor network organization, providing a plausible basis for the observed reductions in pain and enhancements in motor control.

The point selection in this study, particularly Shixuan (EX-UE11), is consistent with both classical indications and modern sensory neuroanatomy. Shixuan points, located at the fingertips, lie within areas of exceptionally high mechanoreceptor density. Stimulation at these sites generates strong afferent volleys to somatosensory networks. High-field fMRI studies confirm that fingertip stimulation activates sharply defined somatotopic representations in S1 regions (areas 3b/1/2), highly responsive to use-dependent plasticity after cortical injury [21,22]. Such sensory-driven cortical activation likely “primes” the motor network for subsequent physiotherapy, increasing cortical excitability and enhancing the efficiency of motor learning. This mechanism aligns with recent computational and behavioral models that show that increased sensory input facilitates error-driven recalibration and fine motor control in neurorehabilitation [23]. In line with these mechanistic considerations, the point selection used in the present study, particularly the inclusion of Shixuan (EX-UE11), appears to be of specific relevance, as improvements in the FMA-UE motor function score were most pronounced in the hand subscale, where a statistically significant between-group difference was observed. Taken together, these findings indicate that Shixuan points likely represent key drivers of the therapeutic effect, specifically by facilitating distal sensorimotor integration and hand motor recovery.

The significant reductions in joint pain are of particular therapeutic relevance. Pain contributes to maladaptive co-contraction, reduced stretch tolerance, and impaired voluntary drive. Thus, pain relief is not simply parallel to symptom improvement but is a likely mediator of motor gains in the acupuncture group. Clinical evidence supports this interpretation, as pain reductions in post-stroke rehabilitation correlate with improved motor performance and functional participation [24]. In this study, improved pain scores may have enabled greater engagement in stretching and more effective activation of voluntary pathways.

The absence of between-group differences in passive joint motion is expected because passive mobility is constrained primarily by structural changes-muscle shortening, collagen crosslinking, and periarticular fibrosis-that develop slowly and respond poorly to short-term neuromodulatory interventions. Similar patterns have been observed in other rehabilitation studies, in which active function improved despite minimal changes in passive range of motion [25]. By contrast, active motor gains reflect neural plasticity and are therefore more sensitive to combined sensory stimulation and motor training.

Although retrospective and non-randomized, the study benefits from its naturalistic setting, which captures routine clinical practice without the behavioral reactivity common in prospective trials. Including all consecutive eligible patients reduces selection bias, and using the validated FMA-UE provides a reliable outcome measure. The sequencing applied here, acupuncture followed by stretching, aligns with evidence that sensory stimulation can transiently increase motor cortical responsiveness, thereby optimizing the effectiveness of subsequent motor training [26]. Patients with substantial pain, intolerance to antispastic medications, or plateaued progress in physiotherapy may derive particular benefit.

Furthermore, Article 9(2)(j) of the GDPR permits the processing of health data for scientific research purposes when adequate safeguards are in place (European Parliament & Council, 2016) [27]. Similarly, the Gesundheitsdatenschutzgesetz Nordrhein-Westfalen (GDSG NRW), §§ 6 and 9, provides that the secondary use of anonymized health data for research is permissible without explicit patient consent, provided that re-identification is not possible (Landtag NRW, 2019) [28]. Because the dataset was irreversibly anonymized, no written informed consent was required, and no formal approval by an external ethics committee was mandated. This interpretation is consistent with international guidance, which states that research on fully anonymized data does not require ethics approval (World Medical Association, 2013; Medical Research Council, 2021) [29].

The study was conducted in accordance with the principles of the Declaration of Helsinki (2013 revision) [30].

Several limitations should be considered when interpreting these findings. This study was a small, retrospective, non-randomized comparative case series, which limits statistical power and increases susceptibility to selection bias, thereby restricting causal inference. The absence of blinding and a sham acupuncture control prevents clear separation of specific acupuncture effects from non-specific contextual or expectancy effects, particularly for subjective outcomes. In addition, potential confounding factors such as differences in concomitant therapies, medication use, or home exercise adherence could not be fully controlled. Finally, the follow-up period was limited to eight weeks, precluding conclusions regarding the durability of treatment effects and long-term functional outcomes. Larger, prospective, randomized controlled trials with blinded assessment and longer follow-up are therefore warranted.

A potential limitation of this study is the baseline difference in time since stroke between the two groups, with substantial variability in Group A. However, all participants were in the chronic phase after stroke (>6 months), a stage in which neurological deficits and post-stroke spastic paresis are generally considered relatively stable. Therefore, although residual confounding cannot be completely excluded, the clinical relevance of this imbalance is likely limited.

The minimal clinically important difference (MCID) for the FMA-UE has been estimated at approximately 9-10 points in stroke rehabilitation. In the present study, both groups exceeded this threshold, indicating that physiotherapy alone already resulted in clinically meaningful motor improvement. This finding is consistent with the established effectiveness of structured rehabilitation programs after stroke. However, the larger improvement observed in the combined treatment group suggests that acupuncture may provide additional benefit when used as an adjunct to physiotherapy. Given the small sample size and retrospective design, these findings should be interpreted cautiously but support further investigation of adjunctive acupuncture in stroke rehabilitation.

Given the small sample size and the exploratory nature of the secondary analyses, findings beyond the prespecified primary outcome should be interpreted with caution.

## Conclusions

Overall, the present findings indicate that acupuncture, when used as an adjunct to physiotherapeutic stretching, may confer additional functional benefits in the management of post-stroke hand spasticity. Compared with physiotherapy alone, the combined approach was associated with greater improvements in upper-extremity motor function, higher overall Fugl-Meyer scores, and a more pronounced reduction in joint pain, whereas changes in passive joint mobility and sensory function were comparable between groups over the eight-week intervention period. The sequence used in this study, acupuncture followed by stretching, is consistent with experimental evidence suggesting that sensory stimulation can transiently enhance motor cortical excitability and thereby optimize the effects of subsequent motor training. Clinically, this approach may be particularly relevant for patients whose rehabilitation progress is limited by pain, intolerance to antispastic medication, or a plateau in conventional physiotherapy.

At the same time, these results must be interpreted in light of the study’s methodological limitations, including its retrospective design, small sample size, and absence of a sham control, which preclude definitive causal conclusions. Nevertheless, the consistency and magnitude of the observed effects suggest a meaningful therapeutic contribution of adjunct acupuncture within routine rehabilitation settings. Taken together, the findings support the potential role of acupuncture as a complementary strategy to enhance standard physiotherapy for post-stroke hand spasticity. Confirmation in adequately powered, prospective randomized trials with longer follow-up is warranted to establish the durability of effects, refine optimal treatment frequency and duration, and identify neurophysiological markers that may help predict treatment responsiveness.
